# Activity *In Vivo* of Anti-*Trypanosoma cruzi* Compounds Selected from a High Throughput Screening

**DOI:** 10.1371/journal.pntd.0001298

**Published:** 2011-08-30

**Authors:** Grasiella Andriani, Anne-Danielle C. Chessler, Gilles Courtemanche, Barbara A. Burleigh, Ana Rodriguez

**Affiliations:** 1 Division of Medical Parasitology, Department of Microbiology, New York University School of Medicine, New York, New York, United States of America; 2 Immunology and Infectious Diseases, Harvard School of Public Health, Boston, Massachusetts, United States of America; 3 Infectious Diseases Unit, Sanofi-Aventis, Toulouse, France; McGill University, Canada

## Abstract

Novel technologies that include recombinant pathogens and rapid detection methods are contributing to the development of drugs for neglected diseases. Recently, the results from the first high throughput screening (HTS) to test compounds for activity against *Trypanosoma cruzi* trypomastigote infection of host cells were reported. We have selected 23 compounds from the hits of this HTS, which were reported to have high anti-trypanosomal activity and low toxicity to host cells. These compounds were highly purified and their structures confirmed by HPLC/mass spectrometry. The compounds were tested *in vitro*, where about half of them confirmed the anti-*T. cruzi* activity reported in the HTS, with IC50 values lower than 5 µM. We have also adapted a rapid assay to test anti-*T. cruzi* compounds *in vivo* using mice infected with transgenic *T. cruzi* expressing luciferase as a model for acute infection. The compounds that were active *in vitro* were also tested *in vivo* using this assay, where we found two related compounds with a similar structure and low *in vitro* IC50 values (0.11 and 0.07 µM) that reduce *T. cruzi* infection in the mouse model more than 90% after five days of treatment. Our findings evidence the benefits of novel technologies, such as HTS, for the drug discovery pathway of neglected diseases, but also caution about the need to confirm the results *in vitro*. We also show how rapid methods of *in vivo* screening based in luciferase-expressing parasites can be very useful to prioritize compounds early in the chain of development.

## Author Summary

Chagas is a devastating disease affecting about 100 million people in Latin America. The drugs available for treatment against the causative agent, the parasite *Trypanosoma cruzi*, have associated toxicity and are not completely effective against the chronic form of the disease, which is the most common presentation in the clinic. There is a great need for new drugs against this disease. Novel technologies in drug development are now being applied for the search of new compounds against Chagas. Taking advantage of a high throughput screening performed recently to identify compounds active against *T. cruzi* replication in host cells *in vitro*, we have selected 23 compounds, which have been re-tested to selected active ones. We have also adapted a transgenic *T. cruzi* expressing luciferase, which allows for direct visualization when mice are infected. These parasites have been used to establish a model for acute Chagas disease useful for drug testing in mice. Using this method, we have tested the activity of the selected compounds and found two compounds with strong anti-*T. cruzi* activity in mice.

## Introduction

It is estimated that around 100 million people live with the risk of infection with *T. cruzi* in endemic areas in Latin America, with approximately 8 million already infected. The considerable influx of immigrants from Latin American countries to USA, Canada and Europe has also made Chagas disease an important health issue in these countries [1].

Although Chagas disease was discovered more than one hundred of years ago, the medicines available for treatment have serious drawbacks. The two drugs current in use, Benznidazole and Nifurtimox that were released in the 70's, present toxic side effects and low efficacy in some strains [2]. It was believed that both of them were only efficient for the treatment of the acute phase but recent studies suggest that chagasic patients in the chronic phase of the disease treated with Benznidazole show reduced disease progression and increased negative seroconversion than the untreated patients [3].

In an advanced position in the pipeline for future anti-*T. cruzi* treatments there is only Posaconazole, an oral antifungal that is currently in the market and has been tested successfully in mice [4] and humans [5] infected with *T. cruzi*. Given the limitations of the current available treatments and the low number of candidates undergoing clinical tests, the development of new anti-*T. cruzi* compounds combining broad and high efficacy with low toxicity is an urgent need.

About ten years ago, the advent of high-throughput screening (HTS) technology revolutionized the process of early drug development, enabling researchers to rapidly collect enormous amounts of data and explore compound libraries with unprecedented thoroughness. Even if this technology has not yielded the expected increase in the number of licencesed medicines in the market, it still is considered a fundamental tool in early drug development in the pharmaceutical industry [6].

Additional developments in the field of drug discovery include luminescent reporter gene assays, which appear as the most prominent type of reporter gene assay used in biomolecular and pharmaceutical development laboratories. The success of these techniques is due to the high signal associated with luciferases, which makes them ideal for high throughput screening (HTS) *in vitro* applications, but also for the possibility of adapting these assays for *in vivo* screening [7].

Major changes are being introduced in the field of Chagas disease drug discovery since the development of recombinant *T. cruzi* parasites to be used as tools for drug screening. The first example is a transgenic *T. cruzi* strain expressing the reporter enzyme β-galactosidase [8] that has allowed performing a HTS for compounds active against *T. cruzi* infection of host cells (Pubchem AID:1885). Screening of drugs in *T. cruzi* mouse models has also been made much more rapid and simple with the use of fluorescent [9] or luminescent [10] recombinant parasites. Recombinant parasites expressing luciferase are already available for several species and have been used effectively for drug discovery in *Leishmania* [11], [12].

In this work we describe the continuation of a chemical HTS against *T. cruzi* trypomastigote infection of host cells. Re-testing of some of the HTS hits for *in vitro* anti-*T. cruzi* activity revealed that approximately half of them did not confirm the activity. Screening of the active compounds in a mouse model of acute Chagas resulted in the finding of one molecular structure with high anti-trypanosomal activity in mice.

## Materials and Methods

### Ethical statement

Animal studies were approved by the Institutional Animal Care and Use Committee of New York University School of Medicine (protocol #81213). This protocol adheres to the guidelines of the Association For Assessment and Accreditation Of Laboratory Animal Care International (AAALAC).

### Compound selection identification and purification

The compounds were selected from a HTS campaign performed by the Broad Institute, as part of the MLPCN (Molecular Libraries Probe Centers Network) *T. cruzi* inhibition project. The results from a HTS for *T.cruzi* trypomastigote infection of host cells were made available at Pubchem (AID: 1885). This HTS was performed by screening of 303,286 molecules (the NIH collection) form where 4,065 hits were selected by their activity against *T. cruzi* trypomastigote infection. These compounds were further assayed to determine their IC50 (Pubchem AID: 2044) and their toxicity to host NIH-3T3 cells (Pubchem AID: 2010).

Compounds were selected from the hits of this HTS among the ones with reported IC50<1.2 µM and at least 100 fold activity versus toxicity All the compounds selected for this analysis had toxicity activity >60 µM. Chromatographic analyses were performed to determine the degree of purification (all compounds were >90% pure except for CID-563075 and CID-2234099 that were 87 and 88% pure, respectively). Electrospray ionization mass spectrometry was performed to confirm compound identification. Finally, compounds were dissolved in DMSO at 10 mM concentration.

### *T. cruzi* and mammalian cells cultures

LLC-MK2 and NIH/3T3 cells were cultivated in DMEM supplemented with 10% FBS, 100 U/ml penicillin, 0.1 mg/ml streptomycin, and 0.292 mg/ml glutamine (Pen-Strep-Glut) at 37°C and 5% CO_2_ atmosphere.

*T. cruzi* parasites from the Tulahuen strain stably expressing the β-gal gene (clone C4) [8] and *T. cruzi* Y strain expressing the firefly Luciferase gene were kept in culture by infection of LLC-MK2 every 5 or 6 days in DMEM with 2% FBS and 1% Pen-Strep-Glut at 37°C and 5% CO_2_ atmosphere.

Trypomastigotes forms were released in the supernatant of infected LLC-Mk2 and harvested between days 5 and 7. The harvested medium was centrifuged for 7 min at 1,237 *g* and, in order to eliminate the amastigotes, the trypomastigotes forms were allowed to swim out of the pellet for at least 3 h. The parasites were counted in a Neubauer Chamber and 10 million trypomastigotes were used to infect 1 million LLC-MK2 cells plated in a 75 cm^2^ culture flask.

### *T. cruzi in vitro* inhibition assay

Between 5 to 7 days after the infection, NIH/3T3 cells and *T. cruzi* Tulahuen expressing β-galactosidase [8] were harvested, centrifuged and washed with DMEM without phenol red supplemented with 2% FBS and Pen-Strep-Glut. The phenol red needed to be eliminated in order to avoid interference with the assay absorbance readings at 590 nM. NIH/3T3 cells (50,000 per well) were seeded in 96-well plates 2 h before addition of purified *T.cruzi* trypomastigotes (50,000 per well) and the compounds for testing at the maximum concentration of 50 µM, therefore the DMSO percentage was never higher than 0.5%. This concentration of DMSO was tested repeatedly and it does not affect the viability of the parasites. Each determination was performed in duplicate. Amphotericin B (Sigma-Aldrich) was used as positive control at a final concentration of 4 µM. Negative and positive controls were carried in every plate. After 4 days, 50 µl of PBS containing 0.5% of the detergent NP40 and 100 µM Chlorophenol Red-β-D-galactoside (CPRG) (Sigma) were added per well. Plates were incubated at 37°C for 4 h and absorbance was read at 590 nm using a Tecan Spectra Mini plate reader.

The absorbance obtained was proportional to the viability of the parasite. The value of IC50 was determined using Graph Prism Software.

### Generation and characterization of luciferase-expressing *T. cruzi* trypomastigotes

The firefly luciferase gene (luc) was used to replace the GFP in the *T. cruzi* episomal expression vector, pTREX-GFP [13], a modified version of pRIBOTEX-GFP [14]. *T. cruzi* (Y strain) epimastigotes maintained at 28°C in LIT medium were transfected with 10 µg of pTREX-luc using a nucleofector transfection system (T-cell protocol; AMAXA) and selected with 200 µg/ml G418 for 4 weeks. Parallel transfections with pTREX-GFP demonstrated that under similar selection conditions >95% of parasites are strongly positive for GFP after 4 weeks (not shown). Mammalian-infective metacyclic trypomastigotes were harvested from stationary phase epimastigote cultures and enriched following passage over DEAE-cellulose/PBS pH.8.0 as routinely performed [15]. Tissue culture trypomastigotes were harvested from monkey kidney epithelial cells, LLcMK2, monolayers infected with Y-luc metacyclic trypomastigotes. Relative luciferase activity in Y strain epimastigotes grown in the presence of 200 µg/ml G418 and in mammalian infective trypomastigotes harvested from infected monolayers after the second passage through mammalian cells (ie. 2 weeks in the absence of drug selection) was similar ∼2.5×10^6^ RLU/10^6^ parasites.

### *T. cruzi in vivo* inhibition assay

Trypomastigotes forms from transgenic *T. cruzi* Y strain expressing firefly Luciferase were purified, diluted in PBS and injected i.p. in Balb/c mice (10^5^ trypomastigotes per mouse). Three days after infection the mice were anesthesized by either i.p. injection of 300 mg/kg of Xylazine and 3500 mg/kg of Ketamine or by inhalation of isofluorane (controlled flow of 1.5% isofluorane in air was administered through a nose cone via a gas anesthesia system). Mice were injected with 150 mg/kg of D-Luciferin Potassium-salt (Goldbio) dissolved in PBS. Mice were imaged 5 to 10 min after injection of luciferin with an IVIS 100 (Xenogen, Alameda, CA) and the data acquisition and analysis were performed with the software LivingImage (Xenogen). One day later (4 days after infection) treatment with compounds at 5 mg/kg/day or vehicle control (DMSO in PBS) was started by i.p. injection in groups of 5 mice and continued daily for the indicated number of days. On the days indicated, mice were imaged again after anesthesia and injection of luciferin as described above. Parasite index is calculated as the ratio of parasite levels in treated mice compared to the control group and is multiplied by 100. The ratio of parasite levels is calculated for each animal dividing the luciferase signal one day after the end of the 5 day treatment (day 9 of infection) by the luciferase signal one day before the beginning of treatment (day 3 of infection).

### Immunofluorescence assay

The compound CID-12402750 was selected for this assay due to its activity against *T. cruzi in vivo*. NIH-3T3 cells plated on coverslips were infected with *T. cruzi* Tulahuen expressing β-galactosidase and incubated with or without drug at 5, 10, 50 or 100 times the value of the IC50 obtained in the *in vitro* assay (IC50 = 0.11 µM). After 3 days, they were fixed with 4% of paraformaldehyde, rinsed with PBS, permeabilized for 15 min in PBS with 0.1% Triton X-100 (Sigma-Aldrich) and blocked for 20 min in PBS with 10% goat serum, 1% bovine serum albumin, 100 mM glycine and 0.05% sodium azide. The cells were incubated for 1 h at room temperature with a polyclonal rabbit anti-*T. cruzi* at 1∶2,000 dilution. After rinsing, they were incubated for 1 h at a 1∶800 dilution with an Alexa Fluor® 488 goat anti-rabbit IgG secondary antibody (Molecular Probes, Invitrogen). DAPI was used to stain the DNA and the coverslips were mounted on Mowiol. Cells were analyzed using an inverted Olympus IX70 microscope with a 60× oil objective.

### Statistical analysis

Data were analyzed using Prism (v. 4.0c, GraphPad). t-test was performed. Statistics were considered significant if *P*<0.05 or *P*<0.01, as indicated.

## Results

The results of the first high throughput screening (HTS) to identify molecules effective against *T.cruzi* trypomastigote infection of host cells were used to select 23 compounds with reported IC50<1.2 µM and at least 100 fold activity versus toxicity for further analysis. The quality control of these compounds was made by HPLC/MASS.

**Figure 1:**
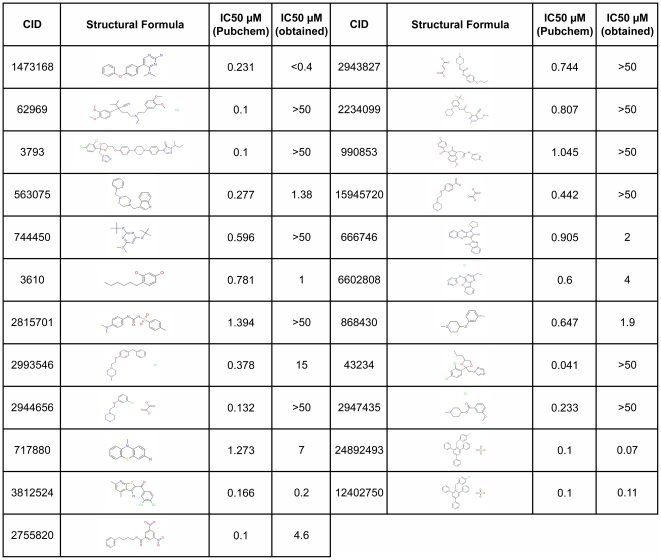
Inhibition of *T.cruzi* growth by 23 compounds selected from the HTS. The IC50 reported from the HTS available at Pubchem and the IC50 determined in our laboratory are shown.

We first tested the activity of the 23 selected compounds against *T. cruzi* trypomastigote infection of host cells using a similar assay and the same parasite and host cells that were used in the HTS (see methods). Our tests showed a higher value for the IC50 for the majority of the compounds when compared to the ones reported in the HTS (Pubchem, AID: 2044), with 11 compounds showing no detectable activity against *T. cruzi* (Fig. 1).

We then selected all compounds that showed activity in our *in vitro* assay for testing of anti-*T. cruzi* activity in mice (except for CID 1473168, which was not available). For this purpose, we adapted a rapid method for drug testing in mouse models using recombinant *T. cruzi* expressing the firefly luciferase gene in an episomal expression vector.

**Figure 2:**
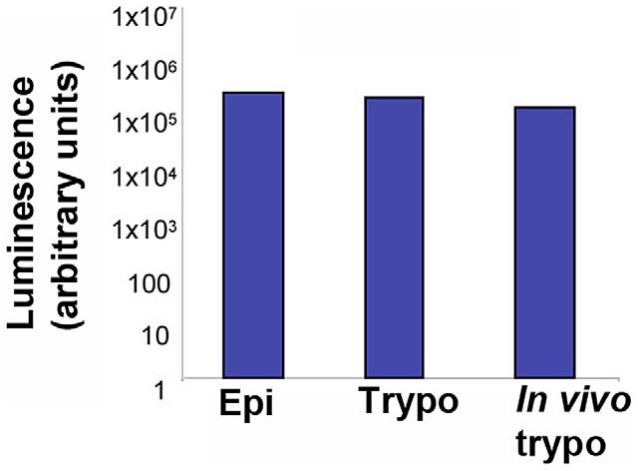
Stable expression of luciferase after *in vitro* or *in vivo* passaging of *T. cruzi* trypomastigotes. Luciferase activity in 1×10^5^ epimastigotes (Epi) under continued drug selection (G418 200 µg/ml) (Epi); 1×10^5^ trypomastigotes (Tryp); 1×10^5^ trypomastigotes 2 weeks after differentiation into metacyclics, removal of drug selection and *in vitro* passage through LLCMK2 cells and subsequent *in vivo* passage in mice where luciferase activity was measured in 10^5^ trypomastigotes acquired from the blood of an infected 6 week-old Balb/c mouse 1 week post infection (*in vivo* trypo).

**Figure 3:**
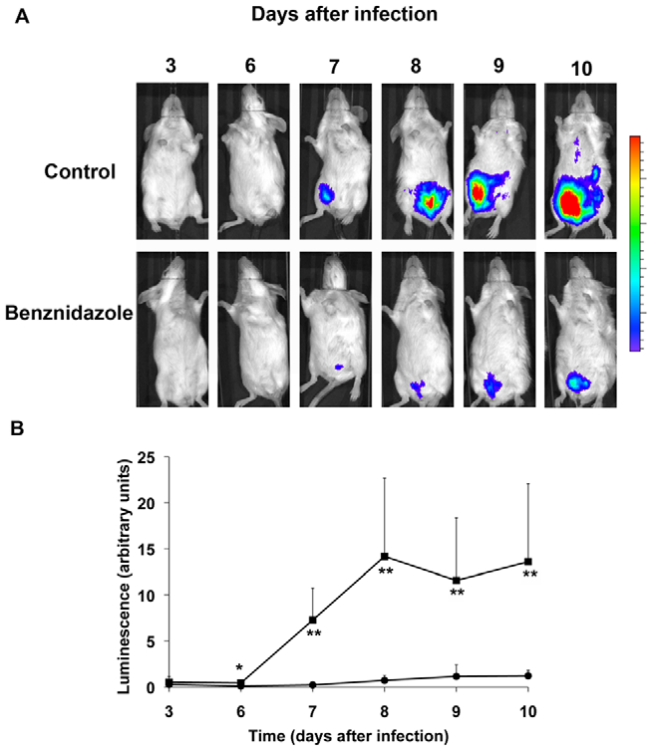
Method for testing anti-*T.cruzi* compounds in mice. Groups of five mice were infected with *T. cruzi* trypomastigotes expressing luciferase and imaged on the indicated days after infection. Treatment with benznidazole (5 mg/kg/day, i.p.) started on day 4. (A) One representative mouse of each group is shown. (B) Quantification of luminescence signal from infected control or benznidazole treated mice. Results are expressed as average ± standard deviation (*, *P*<0.05; **, *P*<0.01).

We generated a recombinant *T.cruzi* expressing luciferase (Y-luc),which presented good infectivity and stability. We find that peak parasitemia was comparable for both WT and Y-luc parasites (data not shown) and that Y-luc trypomastigotes harvested from blood on day 7 of infection exhibited comparable levels of luciferase expression as epimastigotes maintained on drug selection or trypomastigotes that were used to inoculate the mice (Fig. 2). Given that *T. cruzi* amastigotes divide every 12 h and the intracellular infection cycle is 4–5 days, we estimate that these *in vivo* passaged Y-luc parasites were free from drug selection for at least 30 generations (when including time to generate trypomastigotes in culture) [11]. Stable expression of luciferase from pTREX-luc for a minimum of 7 days *in vivo* gives us an adequate window of time to assess the effects of small molecule inhibitors on acute *T. cruzi* infection *in vivo*. Parasite loads were measured at different days after infection by injecting luciferin, the substrate of luciferase, followed by imaging and quantification of the luminescence signal with an IVIS Lumina imager. Focusing on the area of highest intensity signal (red and turquoise), it is clear that luminescent *T. cruzi* are concentrated in the intraperitoneal cavity (site of injection) (Fig. 3A). Following the signal for 10 days post-inoculation demonstrates that there is a clear indication of parasite migration from the injection site (peritoneal cavity) to distal sites, perhaps spleen and liver (Fig. 3A).

Using this recombinant parasite, we infected two groups of five Balb/c mice and followed the course of infection over 13 days. To determine whether this method would be useful for testing of drugs, one of the groups was treated with benznidazole, while control group was injected with vehicle control. A reduced signal was obtained in the group treated with benznidazole (Fig. 3A,B). Even if variation between individual animals is high, as expected in this type of *in vivo* experiments, values between groups are significantly different after only two days of treatment and maintain different levels of infection for the 6 days of treatment.

**Figure 4:**
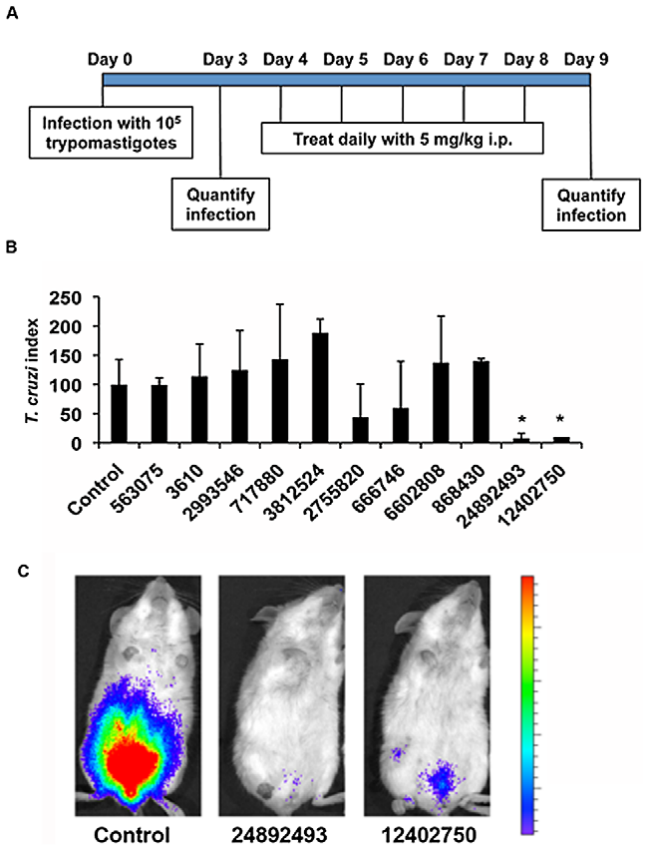
Test for activity *in vivo* of compounds active *in vitro*. Groups of five mice were infected with *T. cruzi* and treated with different compounds following the protocol shown in (A). (B) Quantification of parasite infection levels in the groups of mice treated with the different compounds is expressed as *T. cruzi* index. Compounds are identified by their CID. Results are expressed as average ± standard deviation (*, *P*<0.05). (C) One representative mouse of each group treated with compounds CID-12402750 and CID-24892493.

We then used a modification of this protocol with quantification of the parasite loads only at days 3 and 9 after infection, which corresponds to five days of treatment (Fig. 4A) to test the activity of the eleven compounds selected from the *in vitro* assay (Fig. 1). We found that treatment with some of the compounds had no activity on parasite levels (index close to 100) and others even resulted in increased parasite loads (index higher than 100), possibly because they interfere with the immune response of the mice. However, two of the compounds tested, CID-24892493 and CID-12402750, resulted in severe decreases in the levels of *T. cruzi* in mice that were significantly different from their control group (Fig. 4B,C). No toxic effects were apparent on the mice on visual observation. These two compounds are closely related, they belong to the 1-(4-Halogeno-benzyl)-2,4,6-triphenyl-pyridinium series and are differing only in the nature of halogen on the para position of the benzyl (Fluorine for CID-24892493 and Chlorine for CID-12402750).

**Figure 5:**
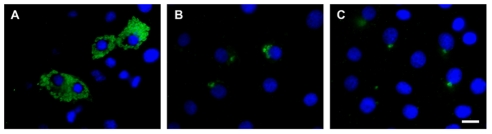
Compound CID-12402750 shows trypanostatic activity *in vitro*. NIH-3T3 fibroblasts were incubated with *T. cruzi* trypomastigotes for 2 h before washing of extracellular *T. cruzi* and addition of drugs. Cells were incubated for 3 days, stained with an anti-*T. cruzi* antibody and DAPI to visualize DNA. Control infection (A) or infection in the presence of compound CID-12402750 at IC5 (B) and IC100 (C) concentration. These are representative images from a total of 50 fields observed in each condition. Scale bar: 10 µm.

To get a better understanding of the anti-*T. cruzi* effect observed, we next determined whether compound CID-12402750 could inhibit *T. cruzi* replication within mammalian host cells. We infected cells for 2 h, rinsed away the remaining free trypomastigotes and, after adding the compound at concentrations between the IC5 and the IC100, we incubated cells for 3 days to allow for amastigote proliferation. In control cells, amastigotes homogenous in size were distributed throughout the cytoplasm of the host cells (Fig. 5A). Treatment with CID-12402750 resulted in infected cells containing only a few amastigotes of average size (Fig. 5B,C), suggesting that this compound interferes with proliferation of amastigotes.

## Discussion

The use of novel pharmaceutical technologies for neglected diseases is opening new possibilities for drug development in this area. As an example of this, the first HTS performed for Chagas disease (Pubchem AID: 1885) represents a major advance in this field. However, our results illustrate the need for confirmation of the HTS results, since as much as 11 hits from the HTS out of 23 selected did not show activity against *T. cruzi* in our hands. Since the same parasite and host cells and were used for the HTS and for testing in our laboratory, and the screening assay is also very similar (see methods), it is likely that the reason for the discrepancies resides in the chemical compounds used for analysis. The quality control of the compounds used in our laboratory was checked by HPLC followed by mass spectrometry and therefore we can be confident that the chemical identity and the purity of the compounds was optimal.

Another recent advance in the drug development field is the development of new methods for screening of compounds in animal models. Testing of compounds for activity in mice was always considered a labor intensive and expensive step in the chain of pre-clinical drug development and therefore was left at the end of the time line. The availability of sensitive imaging techniques and transgenic parasites, either fluorescent or luminescent [9], [10] and the Y-luc parasite described here, allow for rapid testing of relatively high number of compounds. Compared to traditional methods that require bleeding of infected mice and counting parasites in a haemocytometer, intraperitoneal injection of the luciferase substrate and imaging requires considerably less time, with the additional advantage that there is no manipulation of infected blood. Detection of parasites expressing luciferase is also more sensitive than conventional counting of parasites in blood samples. In our model of infection, Balb/c mice infected with *T. cruzi* Y strain, we are not able to detect parasitemia by manual counting in peripheral blood at any time after infection with 10^5^ parasites, but inoculation of the same amount of Y-luc parasites allows to follow the course of infection (Fig. 4). The stability of the Y-luc parasite and the sensitivity of detection allows for screening of compounds early after *in vitro* results, accelerating the speed of pre-clinical drug discovery.

Probably, this method will also be useful in detection of parasitological cure after drug treatments. This is normally achieved by administering an immunosuppressive treatment to mice after drug treatment, when parasites are no longer detectable. If parasites that were not eliminated during the drug treatment emerge after immunosuppression, it is expected that the luminescence signal will still be detectable.

In our project, all the compounds with *in vitro* activity were tested in mice, where we found only one chemical structure with significant *in vivo* activity. It is well known that there is a significant attrition rate when compounds are tested in animal models, even if optimization based on pharmacokinetics parameters has been performed. In our case, direct testing without optimization probably reduces the chances of success, but at the same time, this strategy provides a rapid method to find compounds with activity *in vivo*, which places them in an advanced position in the development chain. Pharmacokinetic analysis can then be performed to optimize compounds accordingly, which combined with testing of anti-parasitic activity *in vivo* in every round, could lead to an accelerated drug discovery path.

We have found a chemical series, 1-benzyl-2,4,6-triphenylpyridin-1-ium, with strong *in vitro* and *in vivo* activity against *T. cruzi*. The two related compounds, with either a Cl or a F in para position of the benzyl, had same level of anti-trypanosomal activity, confirming the efficacy of this structure. We also found that the anti-*T. cruzi* effect is mediated by the inhibition of proliferation of amastigotes within host cells. Despite this promising results, the development of this structure as an anti-trypanosomal drug, may be impaired by the quaternary ammonium, that is generally known as having low intestinal permeability by passive diffusion. Additionally, symmetry, planarity and the presence of 4 phenyl rings could contribute to lower solubility, which would also have a negative impact on oral absorption. Further drug development should include optimization of solubility and permeability *in vitro* before additional tests *in vivo* could be performed.
