# Correction: Activity *In Vivo* of Anti-*Trypanosoma cruzi* Compounds Selected from a High Throughput Screening

**DOI:** 10.1371/journal.pntd.0003293

**Published:** 2014-10-07

**Authors:** The PLOS Neglected Tropical Diseases Staff

The strain of the parasite used in this study appears incorrectly throughout the manuscript. The correct strain should be Brazil strain (Tcl), and not Y strain (Tcll), as described. The corrections and their locations in the paper are outlined below:

In the Methods section, T. cruzi and mammalian cells cultures subsection, second paragraph, the correct sentence is: "…and T. cruzi Brazil strain expressing the firefly Luciferase gene were kept in culture by infection…"

In the Methods section, Generation and characterization of luciferase-expressing T. cruzi trypomastigotes subsection, the correct sentence is: "…T. cruzi (Brazil strain) epimastigotes maintained at…"

In the Methods section, Generation and characterization of luciferase-expressing T. cruzi trypomastigotes subsection, the correct sentence is: "…monolayers infected with Brazil-luc metacyclic trypomastigotes…"

In the Methods section, T. cruzi in vivo inhibition assay subsection, the correct sentence is: "Trypomastigotes forms from transgenic T. cruzi Brazil strain expressing firefly Luciferase…"

In the Results section, paragraph 4, the correct sentence is: "We generated a recombinant T.cruzi expressing luciferase (Brazil-luc)…"

In the Results section, paragraph 4, the correct sentence is: "..for both WT and Brazil-luc parasites (data not shown) and that Brazil-luc trypomastigotes harvested…"

In the Results section, paragraph 4, the correct sentence is: ′…we estimate that these in vivo passaged Brazil-luc parasites were free from…"

In the Discussion section, paragraph 2, the correct sentence is: "…and the Brazil-luc parasite described here…"

In the Discussion section, paragraph 2, the correct sentence is: "…Balb/c mice infected with T. cruzi Brazil strain, we are not able to detect parasitemia by manual counting.."

In the Discussion section, paragraph 2, the correct sentence is: "…but inoculation of the same amount of Brazil-luc parasites allows to follow the course of infection (Fig. 4). The stability of the Brazil-luc parasite…”
